# Supplementary material for: Rapid heating rates define the volatile emission and regolith composition of (3200) Phaethon
Source: Nat Commun. 2024 Aug 21;15:7178. doi: 10.1038/s41467-024-51054-w (PMC11339282; doi:10.1038/s41467-024-51054-w)

Rapid heating rates define the volatile emission and regolith composition of (3200) Phaethon. – **Supplementary Materials**

Suttle, M.D.[1,2], (*corresponding author*), Olbrich, L. F.[3], Bays, C.L.[2,4], Riches, L.[1].
martin.suttle@open.ac.uk, lorenz.olbrich@materials.ox.ac.uk, charlotte.bays@nhm.ac.uk, liza.riches@open.ac.uk.

[1.]School of Physical Sciences, The Open University, Walton Hall, Milton Keynes, MK7 6AA, UK.
[2.]Planetary Materials Group, Natural History Museum, Cromwell Road, London, SW7 5BD, UK.
[3.]Department of Materials, University of Oxford, Parks Road, Oxford OX1 3PH, UK.
[4.]Department of Earth Sciences, Royal Holloway University of London, Surrey TW20 0EX, UK.

Supplementary text

- Extended TGA-MS results
- Supplementary discussion

Supplementary tables

- **Table.S1**. Mass spectrometer ions detected in this study and their inferred parent molecule.
- **Table.S2**. Analyses of Ca-sulphate grains.

Supplementary figures

- **Fig.S1**. Image panel showing TGA-MS data for the main gas phases (H_2O and CO_2) in the five experiments (E, F, G, A and C).
- **Fig.S2**. Image panel showing TGA-MS data for the minor gas phases (SO_2 and C_3H_5) in the five experiments (E, F, G, A and C).
- **Fig.S3**. TGA-MS data for the vacuum heating experiment (sample D).
- **Fig.S4**. Raman spectra showing identification of hematite and Ca-sulphate.
- **Fig.S5**. Evidence of thermal fractures after heating.
- **Fig.S6**. Combined EDX map for sample D illustrating the anticorrelation between S and O abundance, reflecting regions of sulphidation and regions of oxidation within the heated chips.
- **Fig. S7**. Single element EDS maps showing the distribution of sodium in the unheated reference and some of the heated chips.
- **Fig. S8**. Chemical compositions of Ca-rich phases in the experimentally heated samples.
- **Fig.S9**. TGA data from a terrestrial magnetite standard, demonstrating no thermal decomposition under open system heating at temperature <1000 °C.
- **Fig.S10**. Image of the TGA-MS instrument used in this work.

Extended TGA-MS results

Note: see figures S1, S2 and S3 for the complete TGA-MS dataset. Numerical data are presented in Table.2 (found in the main article).

Single cycle, slow rate (Sample E)

Sample E was heated as a single cycle (30 °C – 750 °C – 30 °C) with the slowest heating rate (2 °C/min). This run provides a baseline thermal response of the Murchison meteorite and was designed to aid in the interpretation of more complex heating scenarios and faster heating rates. The total mass loss up to a temperature of 750 °C was 16.1 wt.%. This was composed of H_2O (12.7 wt.%), CO_2 (2.7 wt.%), SO_2 (0.1 wt.%) and organic matter (0.6 wt.%). Water was emitted as three main peaks at <100 °C, at ~410 °C and ~550°C, while the quantity of water emitted at each peak was approximately 4.3 wt.%, 5.0 wt.% and 3.1 wt.% respectively. Carbon dioxide emission occurred primarily at temperatures >600 °C with a peak at ~700 °C, while SO_2 emission occurred in two peaks at ~280 °C and ~520 °C. The emission profile of organic matter peaks at very low temperatures (< 50 °C) and rapidly decreases. At temperatures >100 °C minimal organic matter is released.

Double cycle, intermediate rate (Samples F and D)

Sample F was heated through a double cycle (30 °C – 750 °C – 30 °C – 750 °C – 30 °C) with an intermediate heating rate (10 °C/min). The total mass loss was 14.7 wt.%, with 98 % of the volatile content emitted during the first heating cycle. This was composed of 9.5 wt.% H_2O, 3.6 wt.% CO_2, 1.4 wt.% SO_2 and 0.2 wt.% organic matter. The gas abundance data reveals broader volatile emission peaks which have shifted to higher release temperatures relative to the slower heating experiment E (2 °C/min). This is most notable for the three water emission peaks which remain resolvable but have peak positions approximately 50-100 °C higher (<200 °C, ~450°C and ~620 °C) than the slower heating rate experiment (sample E). Carbon dioxide emission shows a steadily increasing profile at temperatures >200 °C, while SO_2 emission appears as a single peak centred on ~600 °C. Organic matter emission is again dominated by a peak at very low temperatures (< 50 °C), here the decrease in organic release as temperature rises is less dramatic. Above 500 °C minimal organic matter is released.
During the second heating cycle only 0.3 wt.% volatiles were emitted. This was composed of 0.1 wt.% H_2O, 0.1 wt.% CO_2, 0.1 wt.% SO_2 and negligible organic matter. As in the first heating cycle, volatile release continued during the retrograde cooling of the sample.

Sample D was heated under the same conditions are sample F, except that heating occurred under vacuum conditions, instead of within an inert carrier gas (Ar). In the vacuum heating experiment different gas species were not detected, nor were any of the four gas species missing. The data for sample D are similar to sample F in terms of emission quantities. For Sample D the total mass loss was 16.7 wt.% being composed of 16.0 wt.% during the first heating cycle and 0.7 wt.% during the second heating cycle (step 3). Emission profiles suffer from high signal-to-noise ratios due to the lack of the carrier gas.

Double cycle, fast rate (Sample G)

Sample G was heated through a double cycle (30 °C – 750 °C – 30 °C – 750 °C – 30 °C) with a fast heating/cooling rate (20 °C/min). The total mass loss was 11.4 wt.%, with 98 % of the volatile content emitted during the first heating cycle. This was composed of 6.5 wt.% H_2O, 3.9 wt.% CO_2, 0.7 wt.% SO_2 and 0.2 wt.% organic matter. As in sample F the gas abundance data demonstrates broader emission peaks shifted to higher release temperatures relative to both the slow (2 °C/min) and intermediate (10 °C/min) heating rates (Samples E and F respectively). The water emission profile is dominated by a single asymmetric peak with a centre at ~360 °C and shoulders at ~200 °C, ~490 °C and ~650 °C.

Carbon dioxide emission has a smooth profile which rises from ~200 °C and peaks at ~590 °C. Sulphur dioxide emission is negligible at temperatures < 350 °C and peaks at ~460 °C before rising again to a second peak which occurs after the peak temperature (750 °C) was reached, during the retrograde cooling path. The organic matter emission profile is similar to sample F being characterised by a peak at <50 °C and a slow decrease for the remainder of that heating cycle (up to 750 °C).
During the second heating cycle only ~0.3 wt.% volatiles were emitted. This was composed of 0.1 wt.% H_2O, 0.1 wt.% CO_2 and 0.1wt.% SO_2 and negligible organic matter. As in the previous samples volatile release continued during the first half of the cooling window (step 3).

Double cycle, stepped profile, intermediate rate (Sample A)
Sample A was heated through a double cycle with a stepped peak temperature (30 °C – 500 °C – 30 °C – 750 °C – 30 °C) and an intermediate heating/cooling rate (10 °C/min). The total mass loss was 11.6 wt.%, with approximately half (54 %) the volatile content emitted during the first heating cycle, up to T_{max}= 500 °C. Release during the first cycle was composed of 3.6 wt.% H_2O, 1.9 wt.% CO_2, 0.5 wt.% SO_2 and 0.3 wt.% organic matter. By contrast, during the second heating cycle, up to T_{max}= 750 °C released a further 5.0 wt.% volatiles (46.8% of the total volatile content). Release during the second cycle was composed of 2.3 wt.% H_2O, 2.2 wt.% CO_2, 0.5 wt.% SO_2 and 0.3 wt.% organic matter. The gas abundance data reveals pronounced emission peaks in both the first and heating cycle.

For H_2O, emission rose steadily up to ~200 °C before plateauing and then rising again at >250 °C to peak at ~450 °C. On the second heating cycle small amounts of H_2O was released at <500 °C (0.2 wt.%) before rising steadily to a peak at ~630 °C. For CO_2, emission started at >200 °C and rising approximately linearly up to the T_{max} of the first cycle (500 °C). During the second heating ramp negligible CO_2 was emitted at <500 °C. Above this temperature emission plateaued at >600 °C and continued until T_{max} (750 °C). For SO_2, during the first heating ramp emission occurred above ~320 °C and peaked at ~450 °C. During the second heating ramp emissions were again observed as temperatures rose above ~300 °C and were composed of two overlapping events, one smaller peak at ~480 °C and a larger peak centred on ~600 °C. For the organic matter, emission began at 200 °C during the first heating ramp and increased monotonically up to T_{max}. During the second heating ramp emission resumed at ~450 °C and increased up to ~600 °C where emissions then plateaued until T_{max}.

Double cycle, stepped profile, fast rate (Sample C)
Sample C was heated through a double cycle with a stepped peak temperature (30 °C – 500 °C – 30 °C – 750 °C – 30 °C) and a fast heating/cooling rate (20 °C/min). The total mass loss was 11.5 wt.%, with approximately half (61 %) the volatile content emitted during the first heating cycle, up to T_{max}= 500 °C. Release during the first cycle was composed of 4.8 wt.% H_2O, 1.6 wt.% CO_2, 0.4 wt.% SO_2 and 0.3 wt.% organic matter. By contrast, during the second heating cycle, up to T_{max}= 750 °C released a further 4.5 wt.% volatiles (39 % of the total volatile content). Release during the second cycle was composed of 1.8 wt.% H_2O, 2.2 wt.% CO_2, 0.5 wt.% SO_2 and <0.1 wt.% organic matter. The gas abundance data reveals pronounced emission peaks in both the first and heating cycle.

For H_2O, emission rose steadily up to T_{max}= 500 °C during the first cycle and continued to rise in the second cycle only once temperatures exceed 500 °C, peaking at ~650 °C. For CO_2, emission started at >250 °C and rose linearly up to the T_{max} of the first cycle (500 °C). During the second heating ramp negligible CO_2 was emitted at <500 °C. Above this temperature emission plateaued at >650 °C and continued until T_{max} (50 °C). For SO_2, during the first heating ramp a minor emission event was observed at ~200 °C but the main window

of emission occurred above ~300 °C and peaked at ~450 °C. During the second heating ramp emissions were again observed at temperatures above ~300 °C and formed two more distinct events, one centred on ~480 °C and a larger peak centred on ~620 °C. For the organic matter, peak emission occurred at very low temperatures and rapidly decreased. Little organic matter was released at temperatures >100 °C. During the second heating ramp minimal organic matter emission occurred and again, this decreased in abundance as temperatures rose.

The behaviour of the two stepped heating experiments were similar despite differing heating rates.

Repeated heating, 8 cycles, intermediate rate (Sample B)
No TGA or MS data were collected for sample B owing to the length of the experiment. This sample was used only for petrographic study.

Supplementary discussion – Consideration of the Vacuum experiment: Sample F (Ar carrier gas) and Sample D (vacuum) were subjected to the same heating scenario, differing only in ambient gas environments. Both samples exhibited similar behavior in terms of volatile emission as a function of temperature and heating cycle, as well as in total mass loss. However, the average S-depleted rim thickness differed, measuring approximately 170 µm for Sample F and 220 µm for Sample D. This suggests that the vacuum environment allowed gases to escape more readily due to the absence of confining pressure from the flowing Ar carrier phase in the Sample F experiment. Consequently, this effect would have primarily impacted regions near the sample edge, implying that on Phaethon, emissions from the immediate subsurface would be rapid or develop thicker S-depleted zones relative to our experimental runs under an Ar atmosphere.

Supplementary tables

Table.S1. Mass spectrometer ions considered in this study, showing which m/z-values are attributed to which ions and therefore parent molecules.

Parent molecule/s	m/z-value (attributed ion)
H_2O	17 (HO^+), 18 (H_2O^+)
CO_2	22 (CO^{2+}), 44 (CO^{2+})
SO_2	48 (SO^+), 64 (SO^{2+})
Organic matter (C_3H_5)	26 ($C_2H_2^+$), 27 ($C_2H_3^+$), 41 ($C_3H_5^+$).

Table.S2. Representative compositions (EDS spot analyses) of Ca-rich phases in the unheated reference and the experimentally heated samples. Rows are ordered by S abundance and reveal mixed phase compositions between two end-members (calcite and Ca-sulphate). All data are given as uncorrected weight totals (wt.%) and quoted to one decimal place. Low weight totals arise due to the presence of light elements (H, C and O) which cannot be directly detected by EDS. Oxygen abundances were not directly quantified but calculated stoichiometrically (assuming all cations exist in their most common valence state). However, O presence was confirmed by observing the presence of an O-K line in EDX spectra. Under these conditions the ideal stoichiometry for calcite is a weight total of ~56 wt.%.

Sample	Inferred phase	Na	Mg	Al	Si	P	S	Cl	Ca	Cr	Mn	Fe	Ni	O	Total
Sample B	Calcite	-	-	-	-	-	0.1	-	36.1	-	-	0.7	0.1	14.8	51.7
Unheated	Calcite	-	1.0	0.1	1.1	-	0.6	-	34.2	-	-	2.2	0.3	17.2	56.6
Sample D	Calcite	-	-	0.1	-	-	1.4	1.1	35.7	-	-	0.7	0.1	16.7	55.8
Sample C	Mixed: carb-sulp.	-	0.1	0.2	-	0.2	6.1	0.4	33.0	0.1	-	1.8	1.0	23.7	66.5
Sample F	Mixed: carb-sulp.	0.1	1.3	0.2	1.2	-	10.4	0.6	31.4	-	0.1	2.9	0.2	31.5	79.8
Sample F	Mixed: carb-sulp.	0.1	0.3	0.1	0.5	0.2	14.3	0.6	32.7	-	-	1.0	0.2	35.9	85.8
Sample G	Ca-sulphate	-	1.1	0.4	1.3	-	17.6	0.7	29.7	-	0.1	3.8	0.4	42.0	97.0
Sample B	Ca-sulphate	-	0.9	0.5	1.3	-	20.5	0.6	25.7	-	-	6.2	0.1	45.3	101.1

Supplementary figures

Fig. S1.TGA-MS data showing emission profiles for the two major gases (H_2O and CO_2) in each of the five main heating experiments. Data (temperature vs. current [a proxy for the amount of emitted gas]) are split into two columns reflecting the 1st and 2nd heating cycles respectively. Each row shows data for a single experiment. Panels are as follows: (A) Sample E [first heating cycle], (B) no experiment, (C) Sample F [first heating cycle], (D) sample F [second heating cycle], (E) Sample G [first heating cycle], (F) Sample G [second heating cycle], (G) Sample A [first heating cycle], (H) Sample A [second heating cycle], (I) Sample C [first heating cycle], (J) Sample C [second heating cycle]. Note: numbers shown in the top right-hand corner of each plot correspond to the correction factor applied to the Amperes y-axis.

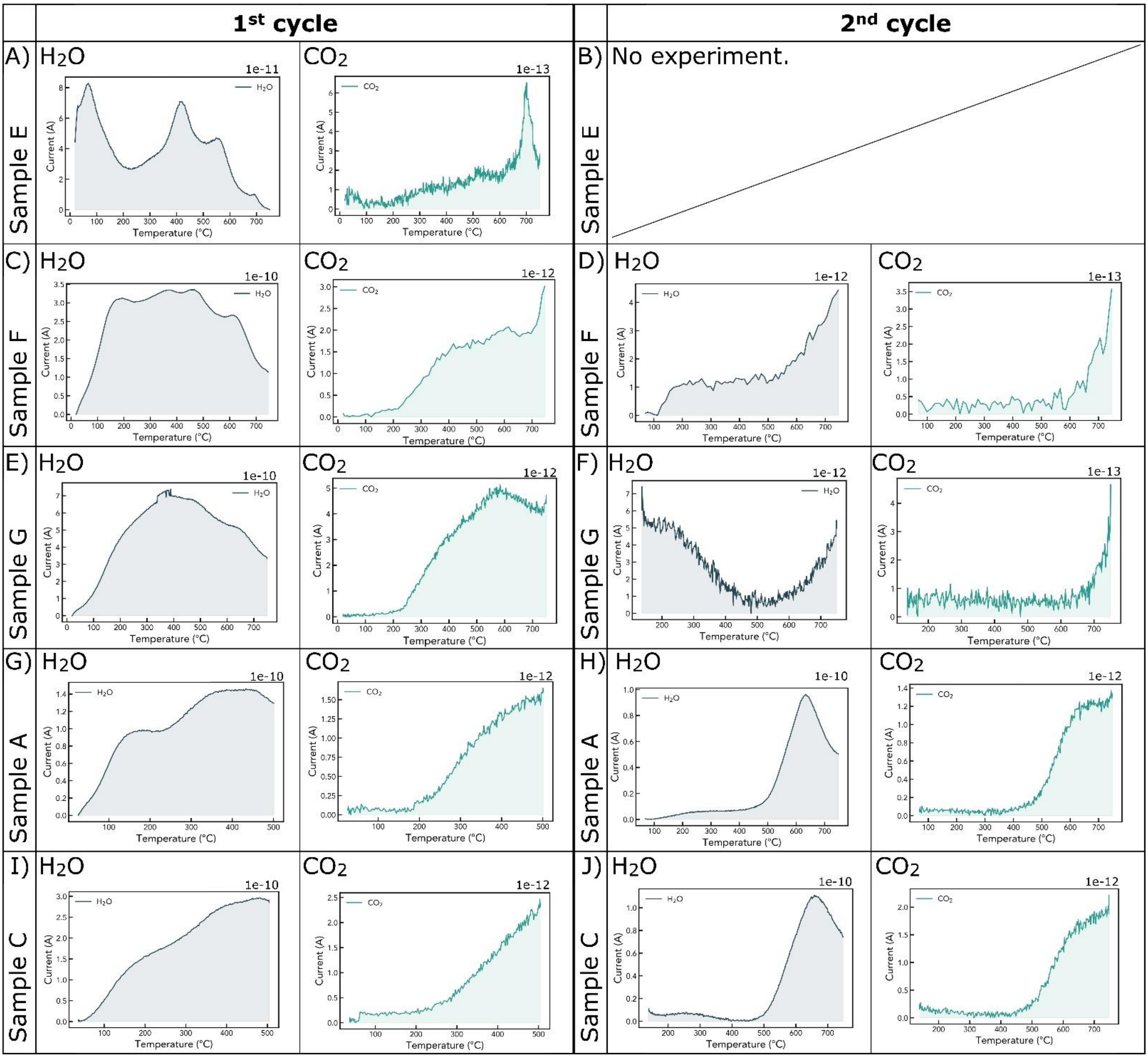

Fig. S2. TGA-MS data showing emission profiles for the two minor gases (SO_2 and C_3H_5) in each of the five main heating experiments. Data (temperature vs. current [a proxy for the amount of emitted gas]) are split into two columns reflecting the 1st and 2nd heating cycles respectively. Each row shows data for a single experiment. Panels are as follows: (A) Sample E [first heating cycle], (B) no experiment, (C) Sample F [first heating cycle], (D) sample F [second heating cycle], (E) Sample G [first heating cycle], (F) Sample G [second heating cycle], (G) Sample A [first heating cycle], (H) Sample A [second heating cycle], (I) Sample C [first heating cycle], (J) Sample C [second heating cycle]. Note: numbers shown in the top right-hand corner of each plot correspond to the correction factor applied to the Amperes y-axis.

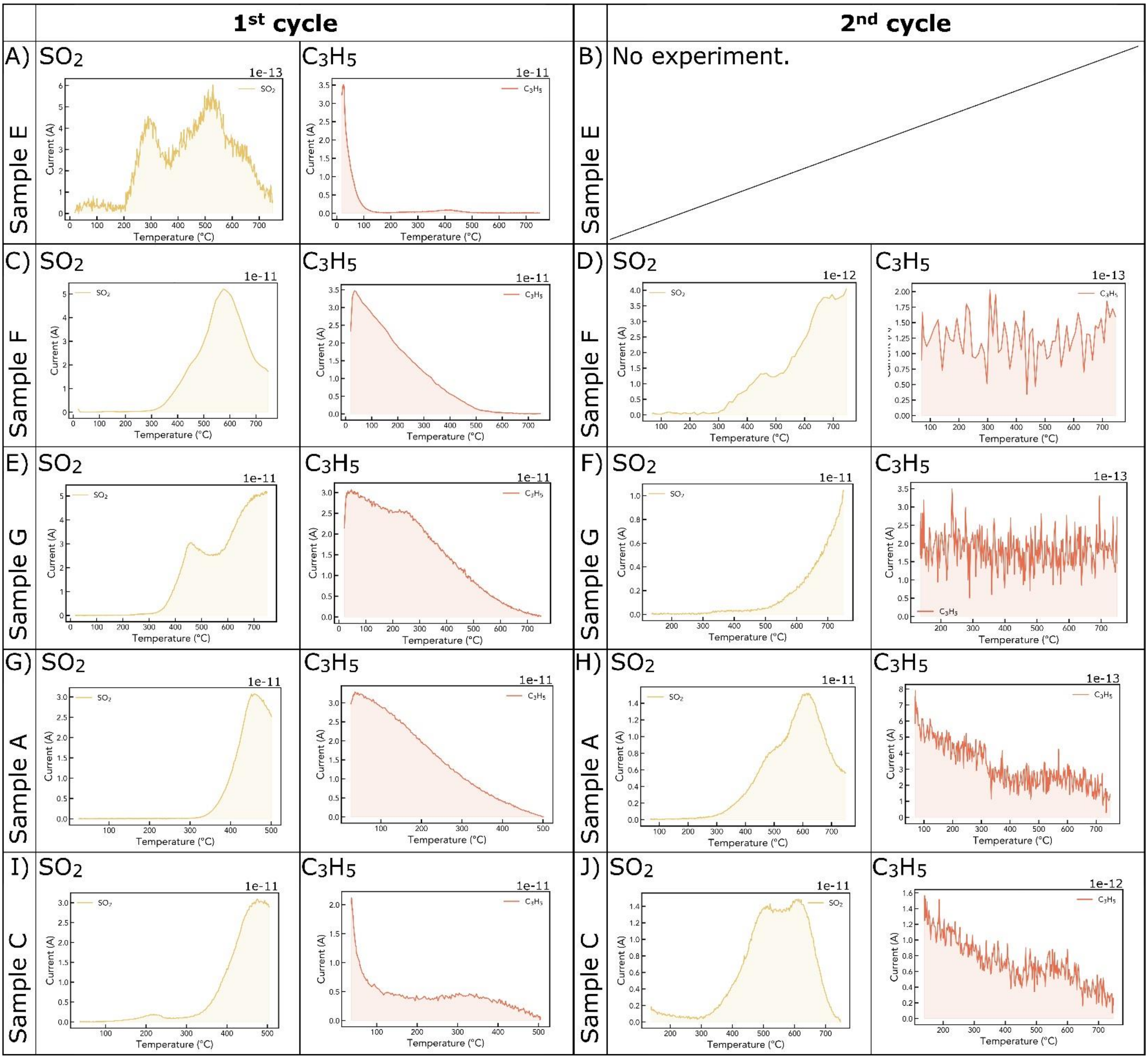

Fig. S3. TGA-MS data for the vacuum heating experiment (sample D – vacuum conditions). These data suffer from low signal-to-noise ratio (noise) due to the lack of a carrier gas in the system during heating. Data are split into two columns reflecting the 1st and 2nd heating cycles respectively. The first row shows the temperature vs. current (a proxy for amount of gas emitted) profiles for the two major gas species (H_2O and CO_2). The second row show the temperature vs. current profiles for the two minor gas species (SO_2 and C_3H_5). Panels are as follows: (A) first heating cycle: H_2O, CO_2, SO_2 and C_3H_5, (B) second heating cycle: H_2O, CO_2, SO_2 and C_3H_5. Note: numbers shown in the top right-hand corner of each plot correspond to the correction factor applied to the Amperes y-axis.

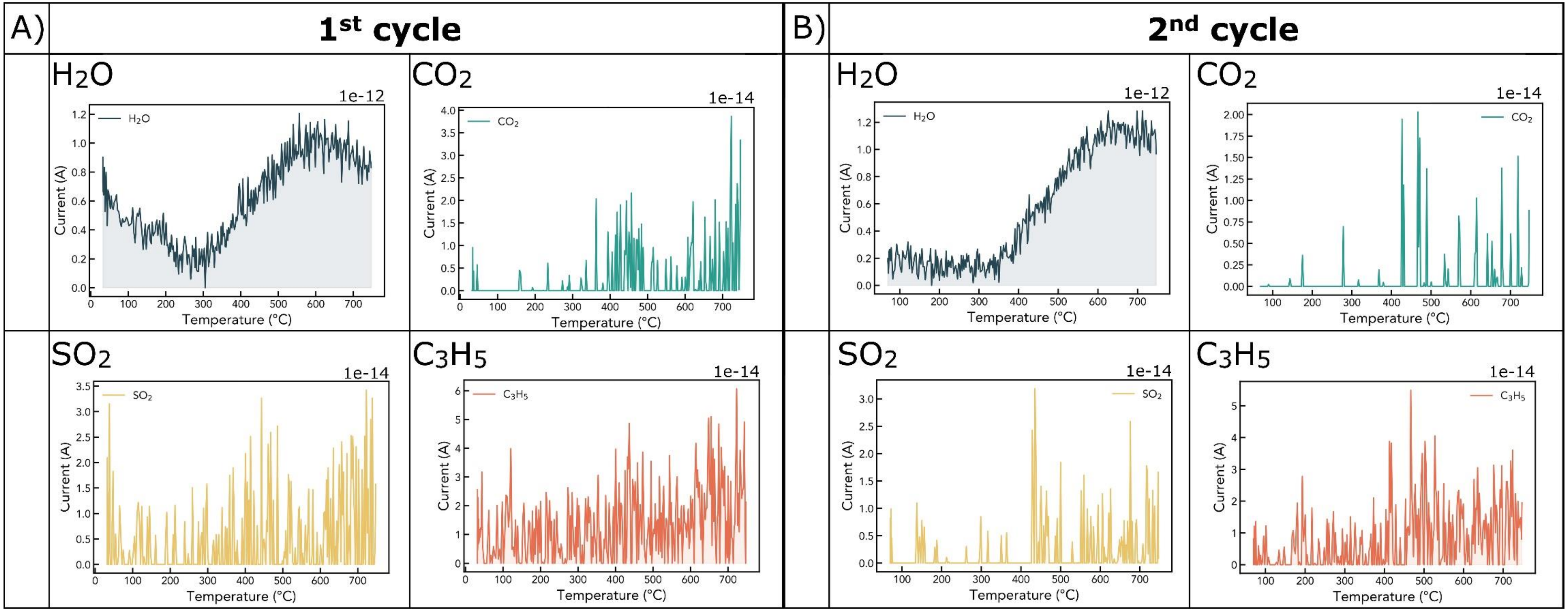

Fig. S4. Raman spectra for mineral identification, confirming the presence of hematite and Ca-sulphate (anhydrite) in the heated samples. Spot analyses within the fine-grained matrix of the heated chips (example here is sample B [shown in grey]) produced spectra containing broad peaks at locations of *228* cm^{-1}, *246* cm^{-1}, *295* cm^{-1}, *413* cm^{-1}, *497* cm^{-1}, *611* cm^{-1} and approximately *1324* cm^{-1}, these correspond to the occurrence of (poorly crystalline) hematite. Spot analyses on the Ca-rich phases (example here is sample B [shown in blue]) returned low quality, noisy spectra with broad peaks. Several of the low frequency peaks correspond to hematite, demonstrating its widespread occurrence throughout the heated chips. However, the strong peaks at *1017* cm^{-1} and the weaker peak at *675* cm^{-1} correspond to the v1 symmetric stretch, and v4 anti-symmetric bending vibrational modes of SO_4 tetrahedra, demonstrating the presence of sulphate minerals. Note, the position of the v1 symmetric stretch mode is dependent on the hydration state of the $CaSO_4$ molecule and varies from *1008* cm^{-1} for gypsum up to *1018* cm^{-1} for anhydrous (anhydrite). Also identifiable in these spectra are broad D (~*1350* cm^{-1}) and G bands (~*1600* cm^{-1}) corresponding to the vibrational excitation of macromolecular organic matter. Reference spectra for hematite (R040024) and anhydrite (R061102) are shown, these data were obtained from the RRUFF database. Note, the spectra shown here been processed for baseline removal, scaling and offset to improve clarity.

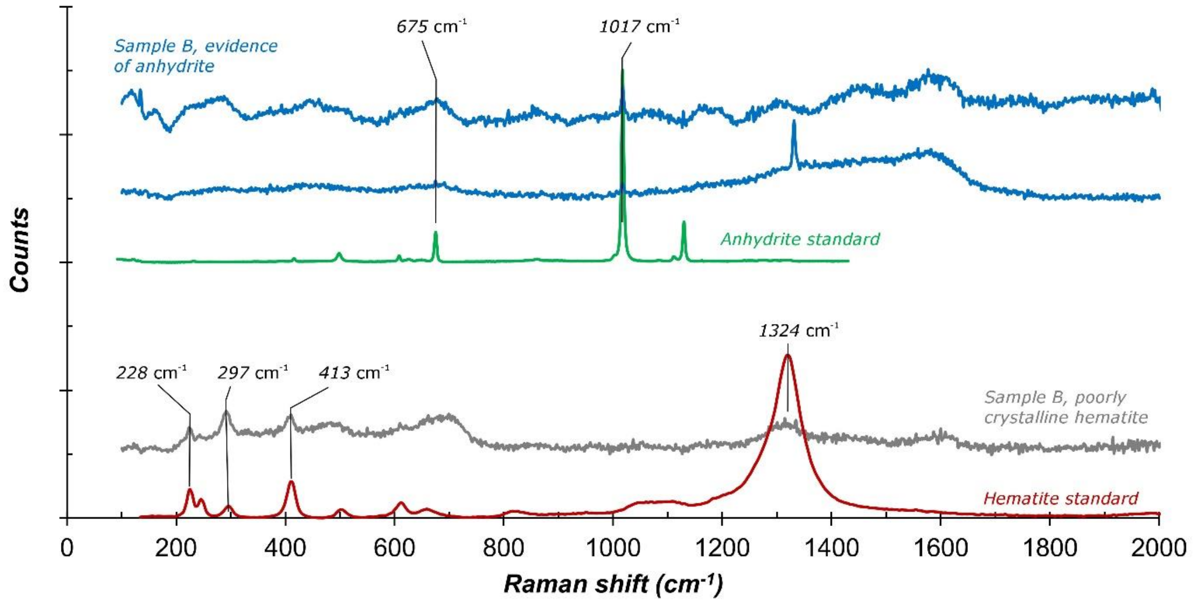

Fig. S5. The texture of the Phaethon analogue evolves with heating. The unheated reference material contains minimal pore space or fractures (A). By contrast the heated samples contain abundant thermal fractures, as demonstrated here from sample A. Radial fractures within chondrule fine-grained rims and penetrating into chondrule cores are common (B).

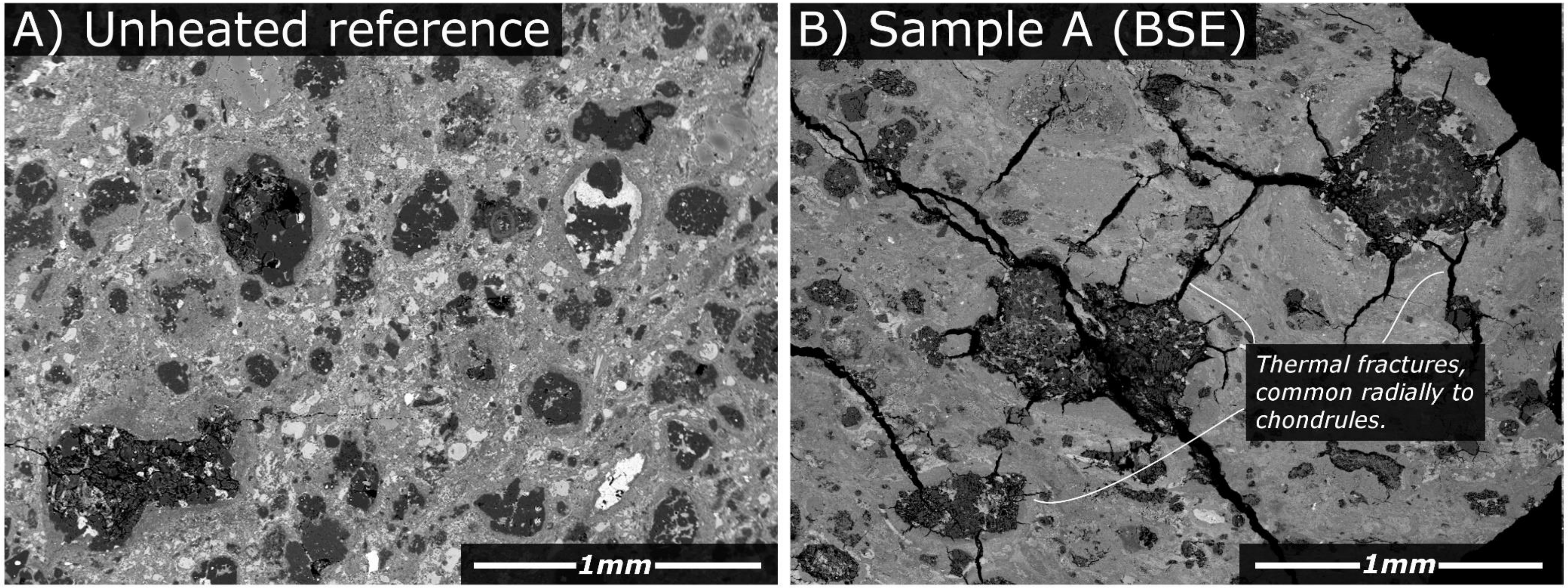

Fig. S6. Combined EDX map (for sample D) illustrating the anticorrelation between S and O abundance, reflecting regions of sulphidation and regions of oxidation within the heated chips. This trend is also present in the other (2x) cyclically heated samples and interpreted as evidence of localised zones where closed system sulphidation dominates and open system gas loss, where oxidation dominates. This leads to Fe-sulphide and Ca-sulphate bearing regions and hematite-bearing regions respectively.

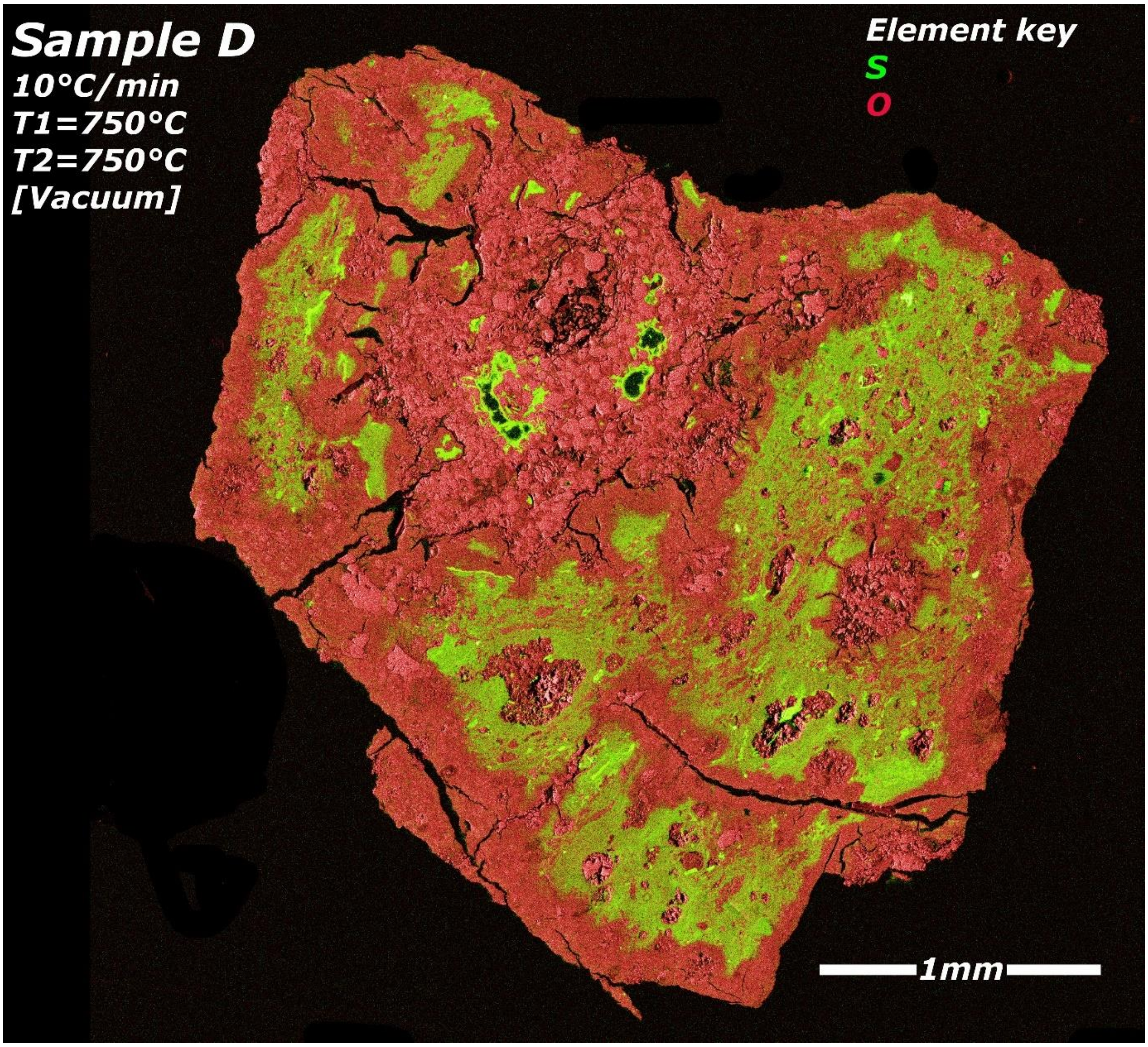

Fig. S7. Single element EDS maps showing the distribution of sodium (Na-K line). Sodium is a minor element in chondritic materials and is homogenously distributed within the fine-grained matrix at the length-scale of a single chip, as demonstrated by the unheated reference material (A). Between chips the absolute Na abundance varies on the order of 0.1-0.6 wt%, reflecting natural variation in Na within the CM lithology. In the twice-heated samples heated samples (B and C) Na has been mobilized, most regions are now depleted, but localised enrichments occur in regions adjacent to chondrules, along fracture walls and along the perimeter of the chip. In the 8x heated sample B (D) Na-distribution is again homogenous (similar to the unheated chip), interpreted as having been mobilized and lost form the sample (evaporative loss).

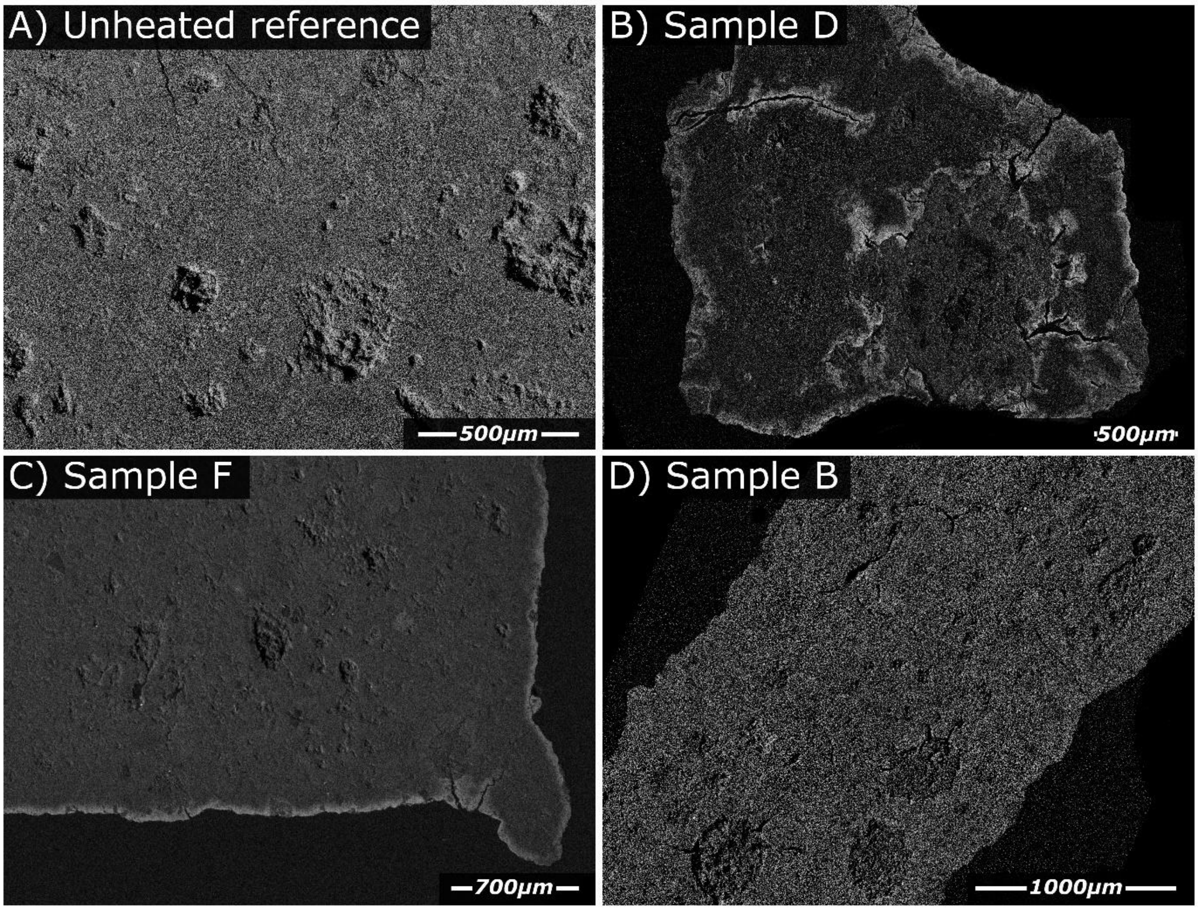

Fig. S8. The compositions of Ca-rich phases in the unheated reference and the experimentally heated samples. This plot compares EDS weight totals (wt.%) against the atomic Ca/S ratios. Calcite is present is the unheated Murchison (black diamonds) but experienced thermal decomposition and reaction with S-gas during the experimental heating to produce Ca-sulphates. Spot EDS analyses on the Ca-rich phases in the experimentally heated samples (red diamonds) return mixed phased compositions reflecting mixtures of two end-member compositions: calcite and Ca-sulphate. Some almost pure Ca-sulphate spots with high weight totals were identified, implying the presence of anhydrite ($CaSO_4$).

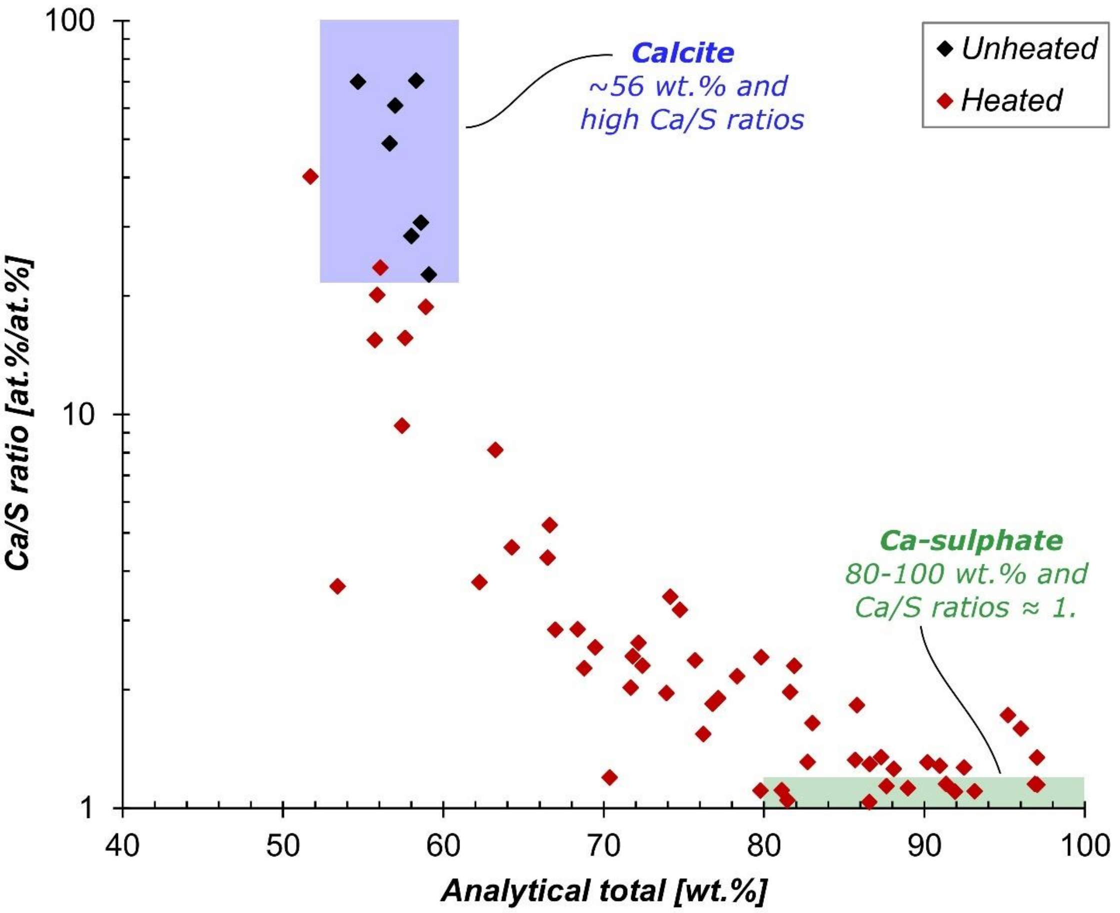

Fig. S9. TGA data from a terrestrial magnetite standard (G1009, obtained from the NHM, London). Data demonstrate that no appreciable thermal decomposition occurs under open system heating conditions at temperatures below 1000 °C (mass loss of <1 wt.%).

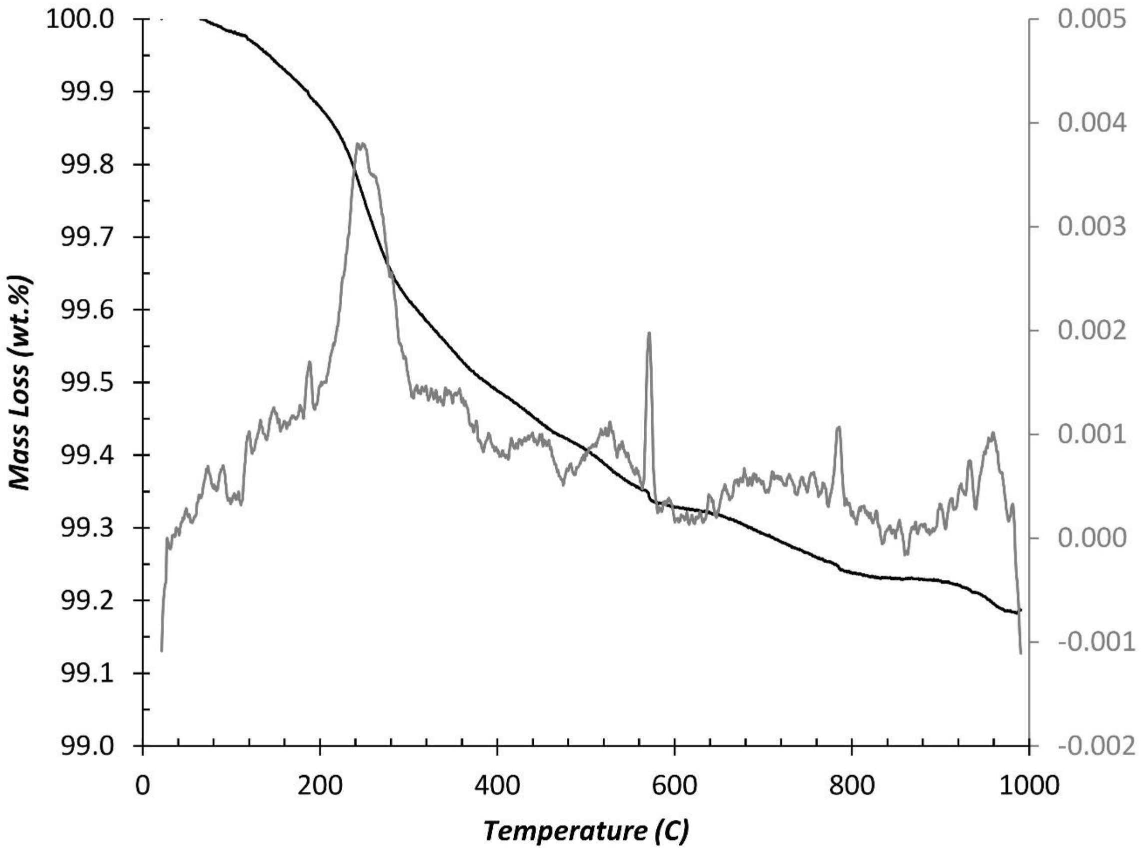

Fig. S10. Image of the TGA-MS instrument used in this work. The exhaust gases from the TGA (a STA 449 F3 Jupiter®, shown on the right) are sent to a mass spectrometer (a QMS 403 Aëolos Quadro, shown on the left). This allows mass loss events, detected in the TGA data to be assigned to specific mineral phase decomposition events. This instrument is based at the Henry Royce Institute, University of Oxford branch, UK.

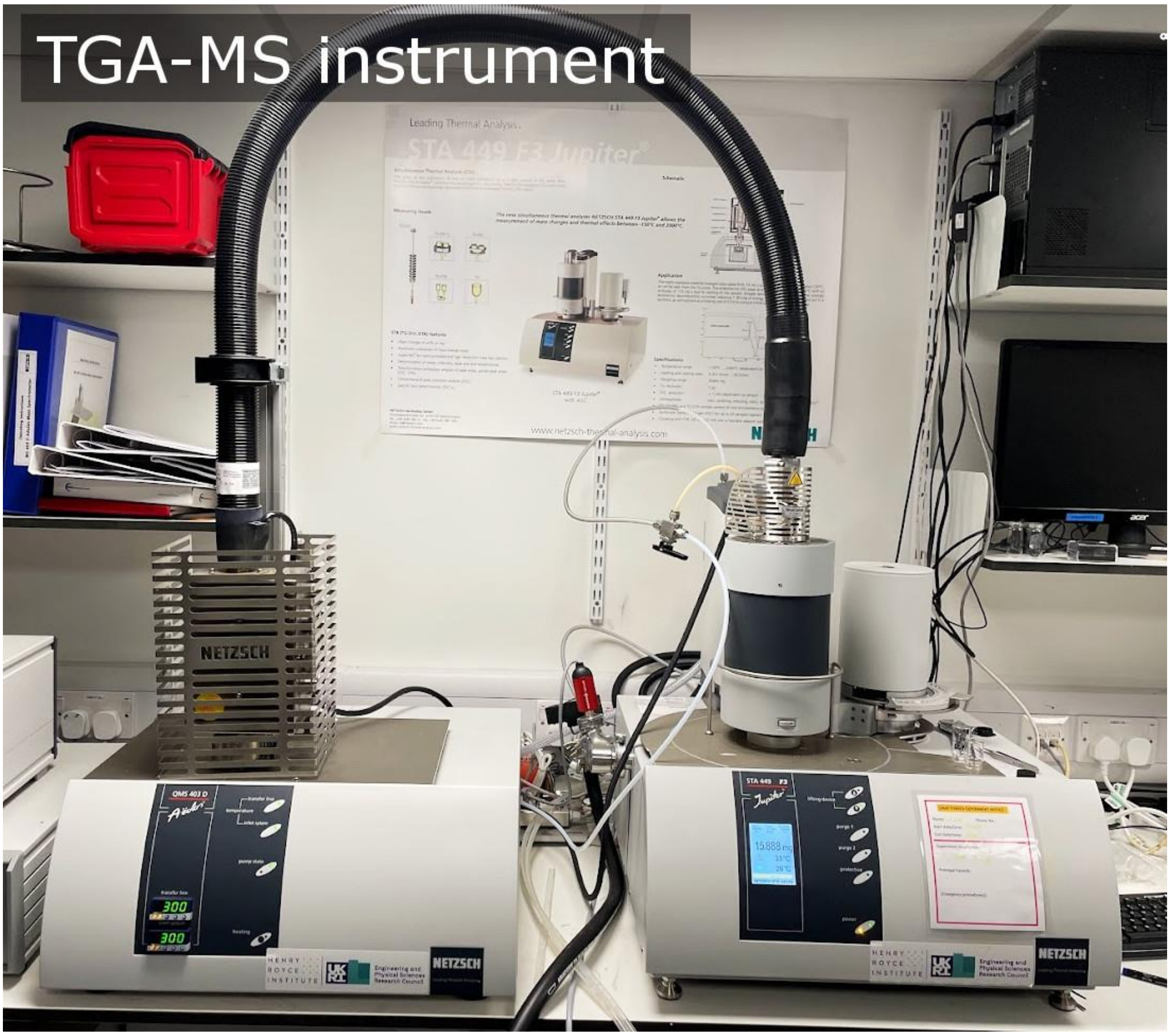

Supplement: Supplementary file 1 — Supplementary Information [file 41467_2024_51054_MOESM1_ESM.pdf]
